# Transient window of resilience during early development minimizes teratogenic effects of heat in zebrafish embryos

**DOI:** 10.1002/dvdy.24640

**Published:** 2018-08-11

**Authors:** Triveni Menon, Sreelaja Nair

**Affiliations:** ^1^ Department of Biological Sciences Tata Institute of Fundamental Research Colaba Mumbai India

**Keywords:** Heat shock, zebrafish, teratogen, tetraploid, centrosome duplication, NEBD, heat resilience, pronuclear fusion, gynogenic diploid

## Abstract

**Background:** Transient heat shock during early development is an established experimental paradigm for doubling the genome of the zebrafish zygote, which has practical applications in expedited identification of recessive mutations in genetic screens. Despite the simplicity of the strategy and the genetic tractability of zebrafish, heat shock has not been used for genome doubling since the proof‐of‐principle experiments done in the 1980s. This is because of poor survival of embryos that ensue from transient heat shocks and gross developmental abnormalities in the few survivors, which is incompatible with phenotype driven screens. **Results:** We show that heat shocks during early zebrafish development uncouple the second cycle of DNA and centrosome duplication. Interestingly, the developmental time of the heat shock that triggers the dissociation between DNA and centrosome duplication cycles significantly affect the potential of embryos to survive and attain normal morphology. The potential to develop normally after a heat shock alters in a developmental time span of 2 min in zebrafish embryos, a phenomenon that has not been reported in any species. **Conclusions:** The existence of heat resilient developmental windows and reduced heat teratogenicity during these windows could be an effective step forward in practical application of transient heat for experimental manipulation of ploidy in zebrafish. More broadly, heat resilience before zygotic genome activation suggests that metazoan embryos may possess innate protective features against heat beyond the canonical heat shock response. *Developmental Dynamics 247:992–1004, 2018*. © 2018 Wiley Periodicals, Inc.

## Key Findings

- Zebrafish embryos at the end of pronuclear fusion and before initiation of zygotic mitosis are resistant to teratogenic effects of heat.
- The teratogenic heat resilient window exists transiently during the maternally controlled phase of development.
- Heat shock during the teratogenic heat resilient window enables generation of morphologically normal zebrafish tetraploids.
- Diploidization of haploids by transient heat shocks during the teratogenic heat resilient windows aids in effective generation of gynogenic diploids.

## Introduction

Mammalian embryos exposed to nonphysiological temperatures either directly or by maternal hyperthermia due to febrile illnesses develop severe developmental abnormalities, including central nervous system defects (Edwards et al., 1997b; Chambers et al., 1998; Graham et al., 1998; Moretti et al., 2005). Embryos of endothermic, ectothermic, and oviparous species are also subject to rising environmental temperatures with dramatic developmental and ecological consequences (Gendelman and Roth, 2012; Rosa et al., 2014; Levy et al., 2015; Griffith et al., 2016). Studies on teratogenic effects of heat in mammals have shown that embryos are heat susceptible early and acquire resilience at later developmental stages (Edwards et al., 1997a; Walsh et al., 1997; Hansen, 2009). The acquisition of heat resilience coincides with the ability to evoke a protective heat shock response after the zygotic genome becomes transcriptionally active (Edwards et al., 1997a; Walsh et al., 1997). In mammals, studies on teratogenic effects of heat involve heat exposure of zygotes for several hours and focus on implantation rates and phenotypic abnormalities in fetuses, which manifest days afterward (Hansen, 2009).

In zebrafish, it is known that a transient heat shock of a couple of minutes during early embryogenesis results in developmental abnormalities. Despite this, transient heat shocks have been shown to be an effective genome doubling strategy in zebrafish (Streisinger et al., 1981). The practical utility of experimentally doubling ploidy in a genetically tractable vertebrate such as zebrafish lies in the expedited identification of zygotic and maternal recessive mutations (Streisinger et al., 1986, 1981; Beattie et al., 1999; Pelegri and Schulte‐Merker, 1999; Pelegri et al., 2004). In zebrafish, a typical F3 screen for zygotic mutations takes ∼6 months and F4 screen for maternal‐effect mutations takes ∼9 months (Driever et al., 1996; van Eeden et al., 1998; Amsterdam et al., 1999; Pelegri and Schulte‐Merker, 1999; Patton and Zon, 2001; Pelegri et al., 2004; Pelegri and Mullins, 2016). However, genome doubling strategies can reduce the mutation screen time by at least one generation wherein heterozygous F2 haploid progeny can be diploidized and the resultant gynogenic diploids in which the mutation is now homozygous, can be directly screened for recessive zygotic mutations, or raised to adults and the F3 embryos can be screened for maternal‐effect mutations (Streisinger et al., 1986, 1981; Beattie et al., 1999; Pelegri and Schulte‐Merker, 1999; Pelegri et al., 2004; Trede et al., 2008).

In zebrafish, experiments done in the 1980s showed that it was possible to double the genome by either perturbing meiosis completion in the egg or by perturbing the first mitosis by applying transient pressure or heat, which disrupts the early embryonic microtubule cytoskeleton (Marsland, 1951; Dasgupta, 1962; Tilney et al., 1966; Streisinger et al., 1986, 1981; Bourns et al., 1988). Perturbing the first mitosis results in a predictable 50% homozygosity in the gynogenic diploid of any locus that is heterozygous in the F2 female (Streisinger et al., 1981; Pelegri and Schulte‐Merker, 1999). In terms of ease of experimental technique, the transient pressure paradigm requires the use of a pressure chamber, whereas heat shocks can be done using a simple water bath immersion method. However, despite the simplicity of experimental procedure and predictable homozygosity efficiency, genome doubling strategies in zebrafish and *Xenopus* use pressure as the experimental paradigm of choice (Pelegri et al., 2004; Noramly et al., 2005; Goda et al., 2006; Trede et al., 2008).

The major limitation in the practical application of transient heat shock as a genome doubling strategy is the early embryonic lethality, which limits the pool of F2 gynogenic diploid embryos for zygotic phenotype screens or for raising to adulthood for maternal screens. In diploid zebrafish embryos exposed to transient heat, embryonic lethality after genome doubling can occur early by ∼24 hr postfertilization (hpf) or later by ∼4–5 days postfertilization (dpf). The late larval lethality is expected because a change in ploidy from endogenous diploidy to tetraploidy is incompatible with survival as adults (Snow, 1975; Eakin and Behringer, 2003). The early lethality in zebrafish is reminiscent of the early embryonic lethality seen in mammalian embryos exposed to nonphysiological temperatures (Edwards et al., 1997b; Chambers et al., 1998; Graham et al., 1998; Moretti et al., 2005). Experiments in mammals show that embryos are heat susceptible early (Edwards et al., 1997a; Walsh et al., 1997; Hansen, 2009). However, to maximize the chances of genome doubling unambiguously in the zygote, zebrafish embryos are subjected to transient heat at the one cell stage, which is a maternally controlled transcriptionally quiescent phase of development. For practical application of the transient heat shock paradigm as a genome doubling strategy, teratogenic effects of heat must be minimized, even when embryos are subjected to transient heat very early in development. We, therefore, wished to ascertain if it was possible to obtain enhanced survival and normal development of zebrafish after a transient heat shock during the first 30 min of development.

Our experiments reveal that there is a transient window of heat resilience in zebrafish zygotes, which is between the end of pronuclear fusion and before the beginning of the first zygotic mitosis. A second transient window of heat resilience occurs during metaphase of mitosis‐I. Both these resilience windows reduce heat teratogenicity in diploids converted into tetraploids and in haploids converted into gynogenic diploids. We have characterized the consequences of the heat shock by analyzing centrosome duplication and nuclear envelope breakdown cycles. Our results show that the cell biological triggers for the diploidization event and eventual survival after transient heat shocks are phenomena that are not coupled to each other.

## Results

### Time of Heat Shock Affects Survival and Potential of Zebrafish Tetraploids to Transition Into Morphologically Normal Larvae

**Figure 1:**
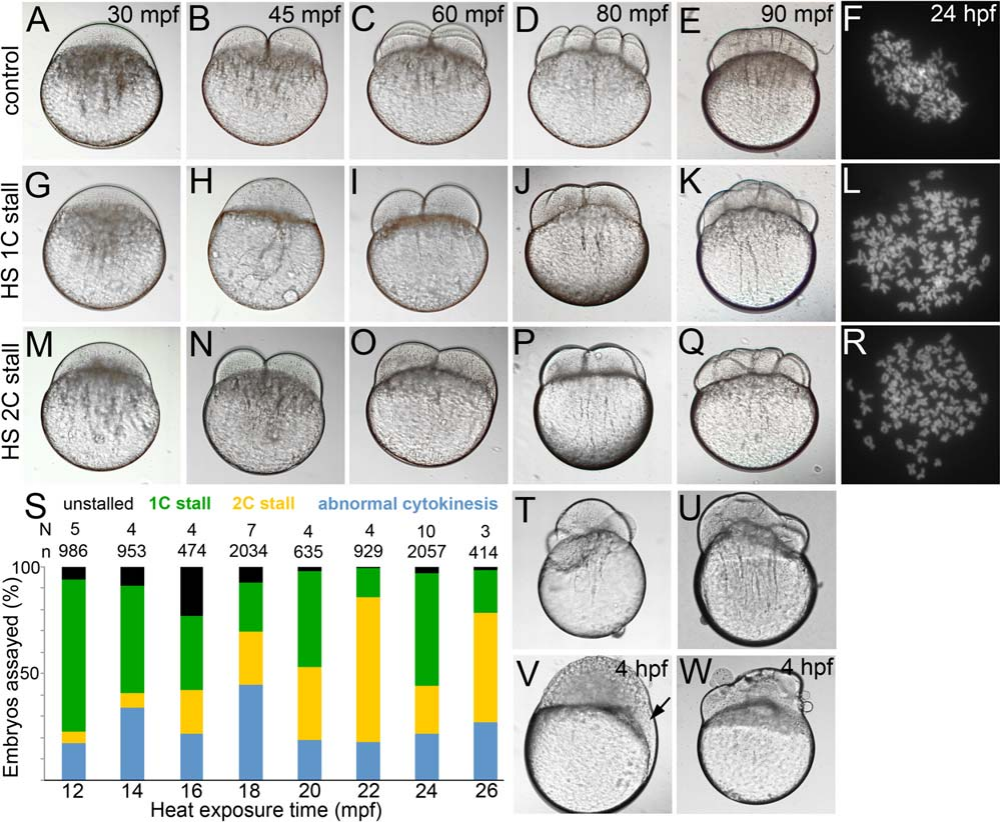
Normal cytokinesis geometries in 1C and 2C stalls. **A–E,G–K,M–Q**: Differential interference contrast images of live control and 1C and 2C stalls from heat shocked embryos, that are offset from controls by one‐cell division cycle each. **F,L,R**: Metaphase chromosome spreads at ∼30 hpf show diploid chromosome numbers in controls and tetraploid chromosome numbers in 1C and 2C stalls. **S**: Categories of embryos obtained across all heat shocks. **T,U**: Embryos undergoing abnormal cytokinesis. **V,W**: Abnormal cytokinesis embryos transition into embryos with acellularized patches (arrow in V) or syncytia.

Zebrafish embryos exposed to ∼2 min of transient heat at 42 °C before the first zygotic division, stall in cytokinesis at either the one‐cell stage (1C stall) or at the two‐cell stage (2C stall) and become tetraploids (Fig. 1A–C,G–I,M–O,F,L,R,S and Heier et al., 2015). In addition to 1C and 2C stalls, early embryonic cytokinesis geometries also deviate from the norm after a heat shock (Fig. 1S–U and Heier et al., 2015). We hypothesized that the embryonic lethality typically associated with a transient heat shock in zebrafish embryos may be due to alterations in cytokinesis geometries, which may culminate in eventual morphological abnormalities and/or lethality. We found that embryos that underwent abnormal cytokinesis geometries frequently transitioned into embryos with acellularized patches in the blastoderm, all of which died during gastrulation or by 24 hpf (Fig. 1V,W). A proportion of heat shocked embryos do not undergo cytokinesis stalls and were not analyzed further (Fig. 1S and Heier et al., 2015).

**Figure 2:**
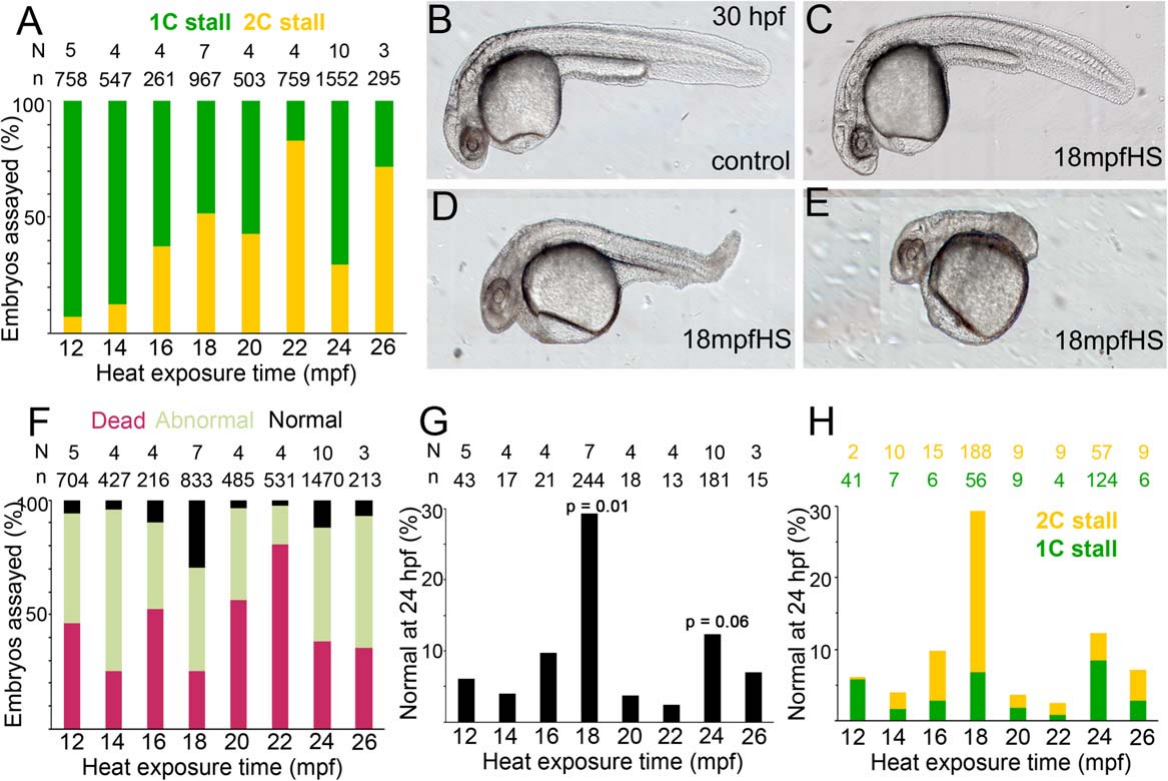
Teratogenic effects of heat and embryonic survival in transiently heat shocked embryos. **A**: 1C vs. 2C stall profile shows a trend to 2C stall in later heat shocks. **B–E**: Live morphology at ∼30 hpf of control and heat shocked embryos show varying degrees of abnormal morphologies upon heat shock. **F**: Embryos with normal cytokinesis geometries and cellularization before epiboly can be categorized into normal, abnormal and dead embryos at ∼24 hpf, with a significant increase in the normal category in 18 mpfHS. **G**: Percentage of embryos with normal morphology across all heat shocks. *P*‐values are for 18 mpf and 24 mpf heat shocks when compared with 12 mpf and were calculated using unpaired *t*‐test and Mann Whitney test. **H**: Contribution of 1C and 2C stalls to total percentage of embryos with normal morphologies in all heat shocks.

A transient heat shock between 12 and 20 min postfertilization (mpf) triggers a predominantly 1C stall behavior whereas those between 22 and 26 mpf, tend toward the 2C stall (Fig. 2A and Heier et al., 2015). In both 1C and 2C stalls, we also found embryos that underwent normal cytokinesis geometries similar to control embryos. We next hypothesized that 1C and 2C stall embryos that underwent normal cytokinesis would survive better and transition into morphologically normal tetraploids. We observed that in addition to the transition in stall from 1C to 2C, the time of heat shock significantly influenced the potential of embryos that underwent normal cytokinesis geometries to survive and develop into morphologically normal tetraploid larvae at 24 hpf. We monitored embryos from the 1C and 2C categories for normal cytokinesis geometries and cellularization before epiboly (Fig. 1A–E,G–K,M–Q). Such embryos from all heat shocks were assayed for survival and morphology at ∼24 hpf. By 24 hpf, heat shocked embryos that underwent normal cytokinesis after 1C or 2C stalls could be sorted as morphologically normal, abnormal, or dead (Fig. 2B–F).

Despite selecting for normal patterns of cytokinesis and cellularization after the heat shock (Fig. 1G–K,M–Q), ∼70% of embryos were either morphologically abnormal or dead by 24 hpf, though all heat shocks did yield some morphologically normal tetraploids (Fig. 2C,F,G). Interestingly, the percentage of morphologically normal tetraploids were significantly higher in clutches heat shocked at 18 mpf, in comparison to those heat shocked at 12, 14, 16, 20, 22, 24, or 26 mpf (Fig. 2C,F,G). As expected, morphologically normal zebrafish tetraploids from all heat shocks died by ∼4–5 dpf and, therefore, tetraploidy per se cannot account for the differential lethality at 24 hpf. Differential survival most likely correlates with developmental processes perturbed at the instant of the heat shock and its recovery once the heat shock is terminated. Because the heat shock was finely tuned to 2‐min intervals, the perturbed processes must be temporally fast for the embryos to recover and resume development after the one‐cycle delay.

### Temporally Dynamic Cell Biological Phases Encompass Transient Teratogenic Heat Resilient Developmental Windows

In newly fertilized zebrafish embryos, the first cytokinesis is morphologically evident by 35–40 mpf with subsequent divisions occurring synchronously at ∼15‐min intervals for the first few cycles (Fig. 1A–E and Kimmel et al., 1995). Mature eggs are arrested at metaphase of meiosis II, which progresses and completes with the extrusion of the second polar body within minutes of the egg coming into contact with water (Selman et al., 1993; Dekens et al., 2003; Nair et al., 2013). If fertilization occurs, egg activation and the initial steps of pronuclear congression and fusion occur concurrently, followed by iterative zygotic mitoses. We first ascertained which early cell biological event was affected by each of the eight heat shocks between 12 and 26 mpf. Control non–heat‐shocked embryos were immunolabeled for α‐tubulin (microtubules; required to execute early cell biological processes) and γ‐tubulin (centrioles and pericentriolar material [PCM]; required for effective organization of microtubules) from 10 to 30 mpf at two minute intervals to span all heat shocks.

**Figure 3:**
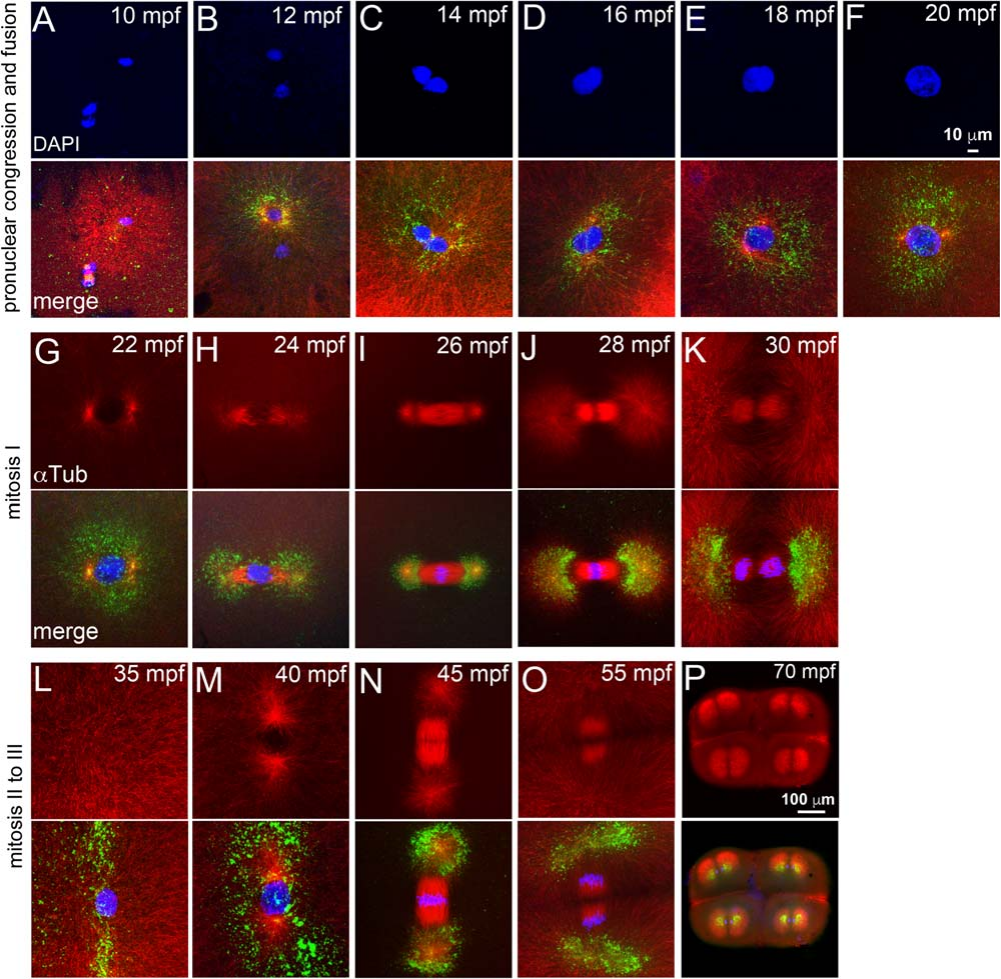
The heat shocks occur during a phase of rapidly evolving cell biology immediately after fertilization. Immunofluorescence labeling of control embryos for DNA (DAPI, blue), α‐tubulin (red) and γ‐tubulin (green). **A–F**: Sperm aster nucleates a microtubule monoaster and the two pronuclei congress to fuse. **G–L**: Mitosis‐I with prophase‐I (G), prometaphase (H), metaphase‐I (I), early (J), late (K) anaphase‐I, and telophase‐I (L). **M–P**: Mitosis‐II with prophase‐II (M), metaphase‐II (N), and anaphase‐II (O). **P**: Mitosis‐III anaphase. In panels L–O, only one of the two cells is shown. Scale bar = 10 μm in F (applies to A–F); 100 μm in P (applies to G–P). Each panel is a representative image of 7 to 10 embryos imaged for a particular time point.

In control embryos, at ∼10 mpf sperm aster microtubule arrays radiated and attached to the female pronucleus initiating pronuclear congression (Fig. 3A). Pronuclear congression continued until ∼16 mpf when the pronuclei came in physical contact with each other triggering fusion (Fig. 3B–D). Pronuclear fusion occurred at ∼18 mpf (Fig. 3E), and the future poles of the first mitotic spindle became evident around the fused pronuclei at ∼20 mpf (Fig. 3F). We considered the appearance of prophase as initiation into mitosis, which begins at ∼22 mpf for mitosis‐I (Fig. 3G), prometaphase‐I at ∼24 mpf (Fig. 3H), metaphase‐I at ∼26 mpf (Fig. 3I), which transitioned into anaphase‐I and telophase‐I by ∼35 mpf (Fig. 3J–L). As the first cytokinesis furrow matured, the second mitosis began with prophase‐II starting at ∼40 mpf (Fig. 3M) and progressing in ∼15 min into mitosis‐III (Fig. 3N–P). Thus, broadly the heat shock could be categorized into three types: those that occurred during pronuclear congression (12, 14, 16 mpf), end of pronuclear fusion (18 and 20 mpf), or the first zygotic mitosis (22, 24, and 26 mpf).

Post heat shock survival and morphology analysis of 1C and 2C stalls that underwent normal cytokinesis patterns and cellularization show that a heat shock at 12, 14, or 16 mpf during pronuclear congression is severely teratogenic and embryonic lethal (Fig. 2F,G). The small proportions of embryos with normal morphology from these categories were tetraploid and died by 4–5 dpf (Fig. 1F,L,R). Similar analysis of 1C and 2C stall embryos from heat shock at 18 mpf revealed an increase in survival and occurrence of morphologically normal tetraploids by up to ∼30% (Fig. 2F,G). Thus embryos exposed to heat at the end of pronuclear fusion and before beginning of zygotic mitosis appear to be comparatively resilient to the teratogenic effects of heat. A heat shock at 20, 22, 24, or 26 mpf during the first zygotic mitosis revealed a reversal in ability to withstand teratogenic heat with the lethality and abnormal morphology fractions resembling a heat shock during pronuclear congression (Fig. 2F,G). Furthermore, we observed that, within mitosis‐I, embryos heat shocked during prometaphase‐I/metaphase‐I (24–26 mpf) tend to survive better (Fig. 2F,G). We tested whether the phenomenon of teratogenic heat resilience during prometaphase/metaphase would hold true for subsequent mitosis in zebrafish by exposing zebrafish embryos to transient heat for 2 min between 40 and 52 mpf, which encompassed prophase to anaphase of mitosis‐II. However, early embryonic lethality was found to be ∼98% in all mitosis‐II heat shocks (data not shown and Heier et al., 2015).

### Cytokinesis Stall Behavior Does Not Influence Eventual Survival and Development of Tetraploid Zebrafish

To understand the differential survival and development into morphologically normal tetraploids, we chose to focus on heat shocks at 12 mpf (12 mpfHS, typically used to generate tetraploids and poor survival), 18 mpf (18 mpfHS, resilient window to teratogenic heat) and 24 mpf (24 mpfHS, potentially resilient window to teratogenic heat during mitosis‐I). Because the transient heat shock also triggered differential cytokinesis stall behaviors among 12, 18, and 24 mpf, we hypothesized that the 2C stall, which occurs in 18 and 24 mpf but not in 12 mpfHS, may be the major contributor to reduced heat teratogenicity at these time points. As expected, parsing the 18 mpfHS and 24 mpfHS that survived to 24 hpf into 1C and 2C stalls revealed that 2C stalls did indeed contribute effectively to embryonic survival and normal morphology; however, 1C stalls also contributed (Fig. 2H). In contrast, the dominant 1C stall in 12 mpfHS is not conducive to embryonic survival (Fig. 2A,H). Thus, the nature of cytokinesis stall per se does not influence the potential of embryos to execute early embryogenesis successfully. Rather, cell biological events that triggered the stall after the heat shock may influence the differential heat teratogenicity observed in 12 mpf, 18 mpf, and 24 mpfHS.

### Transient Heat Shock Before the First Mitosis Uncouples the Second Centrosome and DNA Duplication Cycles in the Zebrafish Zygote

To understand the nature of the cell biological perturbations and recovery upon heat shock, time course immunolabelings were done to assay the microtubule cytoskeleton, DNA and centrosomes after the heat shock. Due to the nature of the experiment, the interval between start of the heat shock and immunolabeling was 6 min (2 min of heat shock + 4 min required to dechorionate and fix embryos). In this study, we considered a pair of centrioles with surrounding PCM as one centrosome.

**Figure 4:**
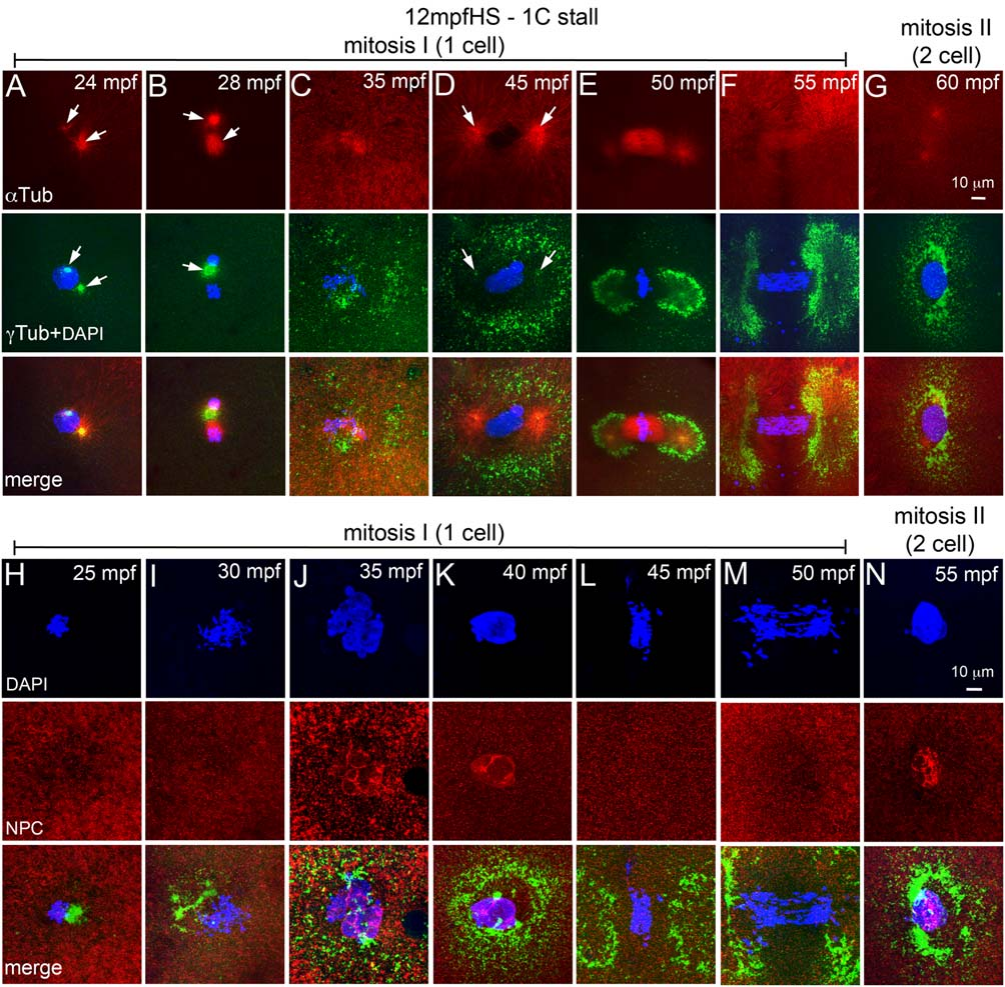
12 mpfHS delays entry into mitosis‐I and does not affect NEBD. **A–G**: Immunofluorescence labeling of 12 mpfHS embryos for DNA (DAPI, blue), α‐tubulin (red) and γ‐tubulin (green). Centrosomes are mispositioned (A,B), PCM recovers (C), and mitosis‐I proceeds with prophase‐I (D), metaphase‐I (E), anaphase‐I (F), and telophase‐I (G). Arrows indicate microtubule asters in the α‐tubulin panels and centrosomal foci in the γ‐tubulin panels. **H–N**: Immunofluorescence labeling of 12 mpfHS embryos for DNA (DAPI, blue), NPC (red), and γ‐tubulin (green). Nuclear envelope does not exist after heat shock at 12 mpf (H,I), is re‐formed at ∼35 mpf (J,K), breaks down upon entry into mitosis‐I (L,M), and re‐forms at the end of mitosis‐I (N). In panels G and N only one of the two cells is shown. For panels A–G, each panel is a representative image of 10 to 15 embryos imaged for a particular time point. For panels H–N, each panel is a representative image of 4 to 6 embryos imaged for a particular time point.

In 12 mpfHS, a single zygotic nucleus and two centrosomal foci were distinctly visible after the heat shock (Fig. 4A). However, unlike control embryos in which centrosomal foci aligned at the opposite poles of the zygotic nucleus in preparation for the first zygotic mitosis (Fig. 3F,G), in 12 mpfHS the centrosome pair was found close to each other (Fig. 4A) or with both centrosomes collapsed into a mass (Fig. 4B). Organization of PCM around the nucleus was completely lost and short astral microtubules nucleated from the mis‐positioned centrosomes (Fig. 4A–C). In control embryos during this phase, a bipolar mitotic spindle assembles to segregate the DNA into daughter cells (Fig. 3H,I). 12 mpfHS embryos recover PCM organization (Fig. 4C) and embryos enter prophase‐I by ∼45 mpf (Fig. 4D). In contrast, control embryos were in prophase‐I at ∼22 mpf (Fig. 3G) and enter prophase‐II at ∼40 mpf (Fig. 3M).

**Figure 5:**
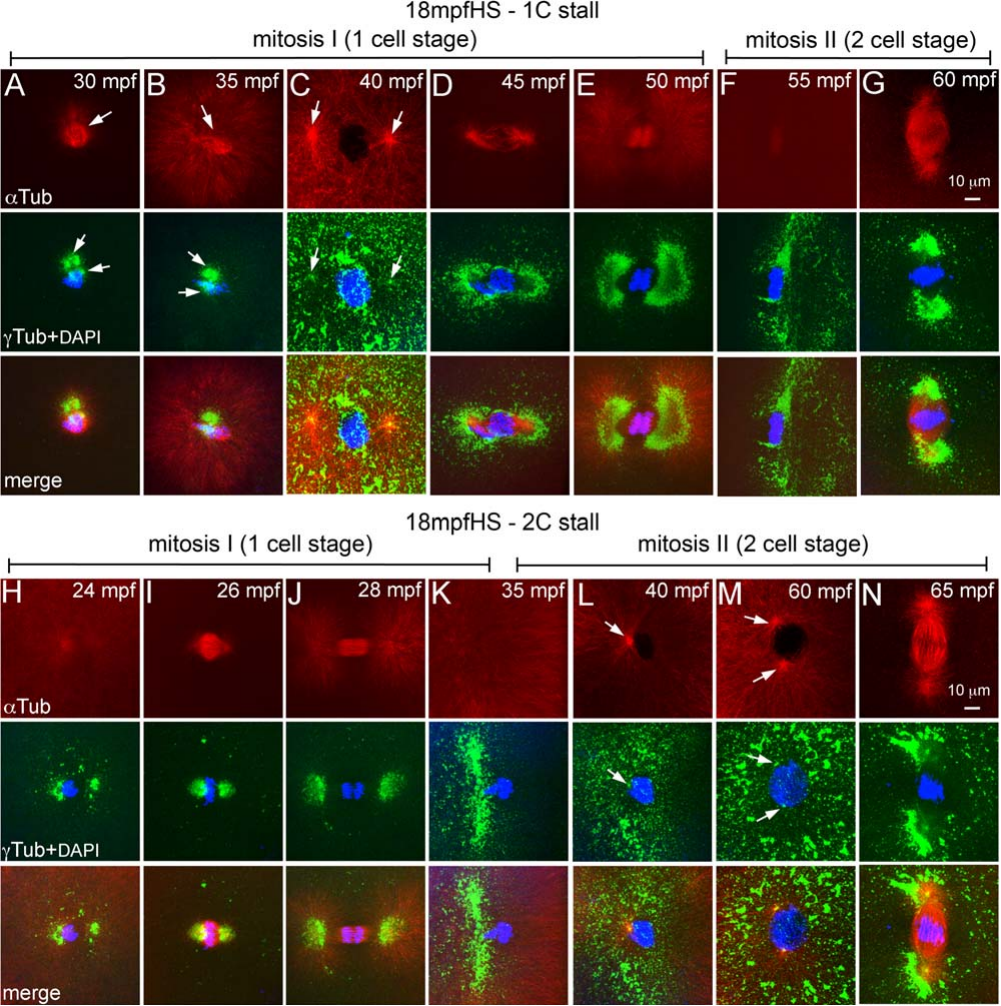
Heat shocks at end of pronuclear fusion perturbs the second centrosome duplication cycle in both 1C and 2C stalls. **A–N**: Immunofluorescence labelings of 18 mpfHS embryos for DNA (DAPI, blue), α‐tubulin (red), and γ‐tubulin (green). A–G: 1C stalls show loss of maternal PCM (A, B), PCM recovery and delayed entry into mitosis‐I (C), and mitosis‐I progression (D–G). H–N: 2C stalls show progression of mitosis‐I (H–K), monoasters in each cell of the two cells (L), subsequent centrosome duplication (M), and entry into mitosis‐II (N). In panels F, G, and K–N only one of the two cells is shown. Arrows indicate microtubule asters in the α‐tubulin panels and centrosomal foci in the γ‐tubulin panels. Each panel is a representative image of 9 to 17 embryos imaged for a particular time point.

By 18 mpf, pronuclei are at the end of fusion phase and embryos are preparing to enter prophase‐I (Fig. 3E,F). 18 mpfHS embryos assayed for microtubules and centrosomes after the heat shock revealed that though most of the PCM was lost upon heat shock, PCM around the two centrosomal foci remained (Fig. 5A,B,H,I). The two centrosomal foci were capable of nucleating microtubule arrays, but the organization of the arrays was dependent on the position of the foci. A collapse of the two foci into a single mass resulted in monoasters (Fig. 5A,B), while bipolar organization of the two foci nucleated mitotic spindles, albeit shorter and stubbier than those in controls (Fig. 5H, I). We categorized the former class of embryos as 1C stall because monoasters will not support progression of mitosis‐I and the latter as 2C stall embryos as they would execute mitosis‐I successfully. By ∼40 mpf, 1C stall 18 mpfHS embryos were in prophase‐I (Fig. 5C), similar to 12 mpfHS embryos (Fig. 4D). Subsequently, these progress through mitosis‐I and enter metaphase‐II by ∼60 mpf (Fig. 5D–G). By ∼40 mpf, the 2C stall 18 mpfHS embryos were in prophase‐II, but with a single centrosome as evidenced by the monoaster (Fig. 5L). Such embryos wait for ∼20 min for centrosomes to duplicate, enter mitosis‐II by ∼60 mpf and are in metaphase‐II by ∼65 mpf (Fig. 5M,N).

**Figure 6:**
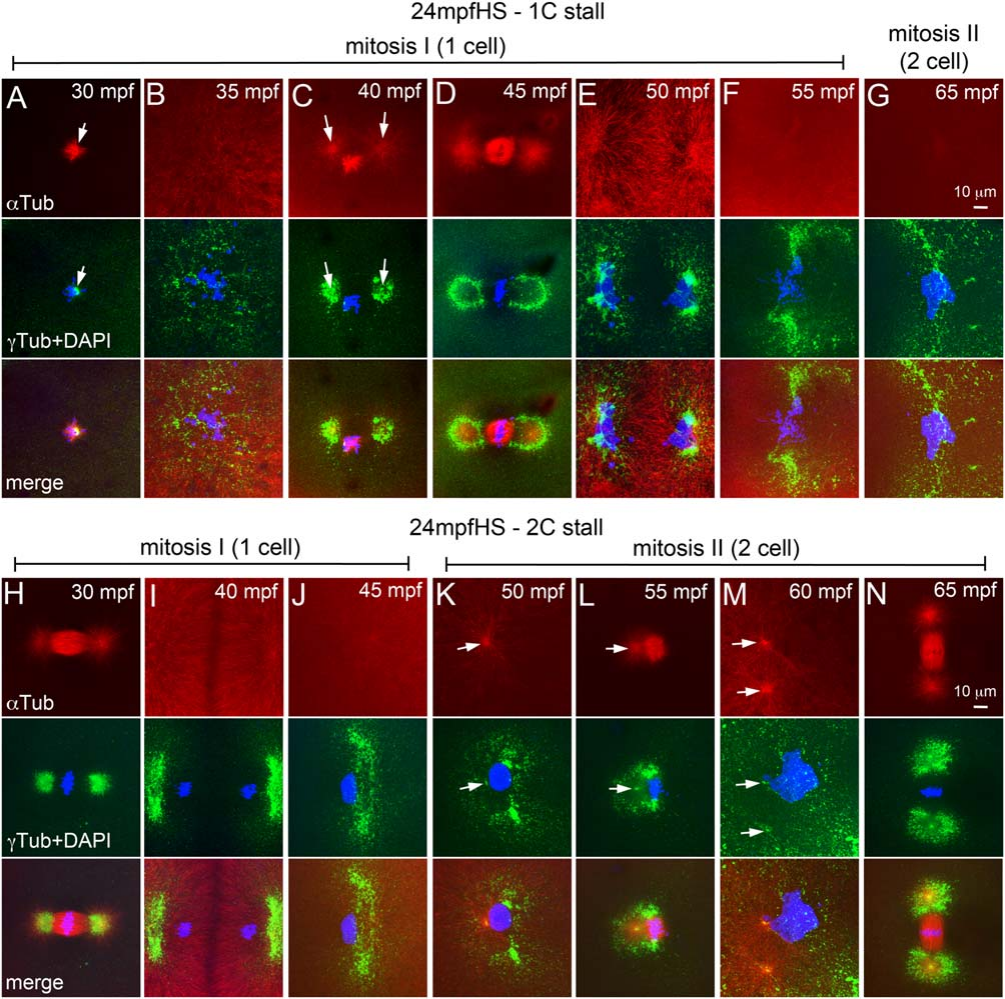
Heat shocks during mitosis‐I also perturbs the second centrosome duplication cycle in both 1C and 2C stalls. **A–N**: Immunofluorescence labelings of 24 mpfHS embryos for DNA (DAPI, blue), α‐tubulin (red), and γ‐tubulin (green). A‐G: 1C stalls show loss of maternal PCM (A), PCM recovery (B), and delayed entry into mitosis‐I (C) and mitosis‐I progression (D‐G). H‐N: 2C stalls show progression of mitosis‐I (H–J), monoasters in each cell of the two cells (K,L), subsequent centrosome duplication (M), and entry into mitosis‐II (N). In panels F, G, and J–N only one of the two cells is shown. Arrows indicate microtubule asters in the α‐tubulin panels and centrosomal foci in the γ‐tubulin panels. Each panel is a representative image of 11 to 28 embryos imaged for a particular time point.

At the 24 mpfHS, embryos are in prometaphase‐metaphase of mitosis‐I (Fig. 3H,I). 24 mpfHS embryos assayed for microtubule cytoskeleton, centrosomes and DNA at ∼30 mpf revealed that similar to 18 mpfHS, most of the PCM was lost and what remained was around the two centrosomal foci (Fig. 6A,H). The two foci were either found as a single mass (1C stall, Fig. 6A) or as a bipolar microtubule organizing center (2C stall, Fig. 6H). PCM organization recovers in 1C stall by ∼35 mpf (Fig. 6B), embryos re‐enter mitosis‐I by ∼40 mpf (Fig. 6C–E) and proceed through subsequent mitoses (Fig. 6F,G). Mitosis‐I completes normally in 2C stall 24 mpfHS (Fig. 6H–J); however, these embryos possess a single centrosome when prophase‐II initiates (Fig. 6K,L), which cannot support mitosis‐II progression. 24 mpfHS embryos also wait until ∼60 mpf for centrosomes to duplicate and enter metaphase‐II by ∼65 mpf (Fig. 6M,N).

### Transient Heat Shocks Do Not Perturb NEBD Cycles in Zebrafish Embryos

Because 1C stall embryos could potentially enter mitosis‐I after recovery of centrosome positioning and PCM organization but do so only close to the next programmed cycle of prophase for mitosis‐II, we reasoned that perhaps another cyclical cell biological event which is unperturbed by the heat shock continues to occur, and this event forces heat shocked embryos to follow an inherent early cell biological program.

**Figure 7:**
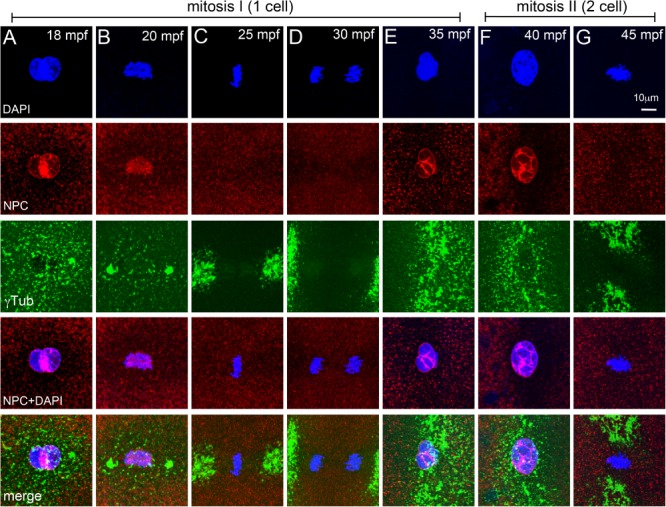
NEBD cycles during early mitosis in zebrafish embryos. **A–G**: Immunofluorescence labelings of control embryos for DNA (DAPI, blue), NPC (red), and γ‐tubulin (green). Nuclear envelope exists around pronuclei (A), disassembles at onset of and during mitosis‐I (B–D), re‐forms during telophase‐I (E), and exists during prophase‐II (F), but disassembles again for mitosis‐II (G). In panels E–G only one of the two cells is shown. Each panel is a representative image of 4 to 6 embryos imaged for a particular time point.

Animal cells undergo open mitosis, which involves break down of the nuclear envelope (NEBD) for each mitotic cycle (Fernandez‐Alvarez and Cooper, 2017). In zebrafish, during late cleavage stages and later in the blastula, nuclear envelope reassembly during mitosis has been studied using a Mab414 antibody which recognizes the FXFG repeats in the nucleoporins p62, p152, and p90 (Abrams et al., 2012). We used the Mab414 antibody to immunolabel zebrafish embryos for the nuclear pore complex (NPC) between 10 and 60 mpf to understand the cyclical nature of NEBD during pronuclear congression, fusion and mitosis (Fig. 7). In zebrafish embryos, nuclear envelope surrounds each pronucleus during congression and fusion phases (Fig. 7A). Co‐incident with entry into mitosis, NEBD occurs between 20 and 30 mpf (Fig. 7B–D).

By 35 mpf, the nuclear envelope re‐forms around each daughter nuclei which are in telophase‐I (Fig. 7E), exists during initiation of prophase‐II (Fig. 7F) and breaks down again by ∼45 mpf during metaphase‐II (Fig. 7G). In 1C stalls we predicted that programmed NEBD cycles if unperturbed by the heat shock, could impede immediate progression into mitosis even after recovery of centrosome positioning and PCM organization. Such embryos would have to wait for the next scheduled cycle of NEBD to enter mitosis, which will occur at ∼45 mpf for mitosis‐II. Assaying for presence of the nuclear envelope in 12 mpfHS embryos (in which 1C stall is predominant) revealed that co‐incident with recovery of PCM organization (Fig. 4H–J), the nuclear envelope also re‐formed by ∼35 mpf (Fig. 4J,K). DNA duplication occurs in both control and 12 mpfHS embryos during this phase, the key difference being that in heat shocked embryos, the DNA of the unsegregated zygotic nucleus duplicates, resulting in a tetraploid genome. Subsequently, NEBD occurs to allow mitosis‐I and subsequent reiterative mitoses to continue (Fig. 4L–N). NEBD cycles also remained unperturbed in 18 mpf and 24 mpfHS and in 1C and 2C stalls (data not shown). Thus, a transient heat shock affects centrosome positioning, microtubule organization, and centrosome duplication but does not perturb NEBD and re‐formation. Normally, each NEBD instance is followed by mitosis, whereas in heat shocked embryos NEBD occurs successively twice before a mitosis.

### Transient Developmental Windows of Teratogenic Heat Resilience Aid in Efficient Production of Gynogenic Diploid Zebrafish

**Figure 8:**
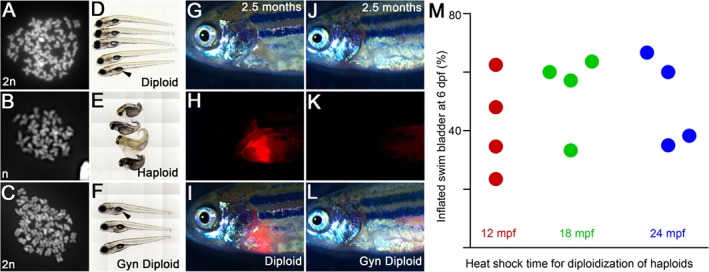
Teratogenic heat resilient developmental windows can be used for production of gynogenic diploids. **A–C**: Metaphase chromosome spreads show that diploids have 50 (24 hpf, A), gynogenic haploids have 25 (48 hpf, B), and heat shocked gynogenic haploids have 50 (48 hpf, C) chromosome counts, indicating a genome doubling event in the haploids upon transient heat shock. **D–F**: Live images at six dpf of diploids (D), haploids (E), and gynogenic diploids (F) show swim bladder inflation in diploids (D) and gynogenic diploids (F), but not in haploids (E). **G–I**: IVF using spermatozoa from *Tg(cmlc2:mCherry)* males result in transmission of the transgene from the male genome into the progeny. **J–L**: IVF using UV irradiated spermatozoa from *Tg(cmlc2:mCherry)* males produces haploids, which after a heat shock become gynogenic diploids with only the maternal nontransgene carrying genome. G,J: brightfield, H,K: cardiac mCherry fluorescence channel, I,L: merge. **M**: Quantification of swim bladder inflation in multiple trials of gynogenic diploids that were generated by transient heat shock of haploids at 12, 18, and 24 mpf.

A major hurdle in the use of transient heat as a genome doubling strategy is the fact that heat is a developmental teratogen. Our experiments unexpectedly revealed that zebrafish embryos are less prone to teratogenic effects of transient heat shocks at 18 and 24 mpf, in comparison to 12 mpf. Although our observations were focused on tetraploid embryos, we wished to additionally ascertain the utility of the teratogenic heat resilience windows of 18 and 24 mpf toward generation of gynogenic diploid embryos in comparison to 12 mpf. We generated gynogenic haploid embryos by in vitro fertilizing eggs using UV‐irradiated sperm solution. Metaphase chromosome spreads showed that such embryos are indeed haploids with a chromosome count of 25 instead of the normal diploid count of 50 (Fig. 8A,B). Between 1 and 5 dpf, gynogenic haploid embryos manifested a cluster of progressively severe developmental abnormalities referred to as the haploid syndrome, which include short body axis, cardiac edema, and microphthalmia (Fig. 8D,E).

Additionally, in comparison with diploid embryos, all gynogenic haploids failed to inflate the swim bladder (Fig. 8D,E) and eventually died. We subjected sister clutches of gynogenic haploid embryos to transient heat shocks at 12, 18, and 24 mpf to diploidize the genome. Metaphase chromosome spreads confirmed that heat shocked gynogenic haploids had undergone a genome doubling event as the chromosome counts were restored to diploidy (Fig. 8C). We also assayed for the absence of paternal traits in gynogenic diploids to confirm that the genome is entirely maternal by using UV‐irradiated spermatozoa from *Tg(cmlc2:mCherry)* (Palencia‐Desai et al., 2011) males. Diploid fish obtained by in vitro fertilization (IVF) of eggs with *Tg(cmlc2:mCherry)* spermatozoa have cardiac mCherry fluorescence, which is contributed only by the paternal genome (Fig. 8G–I). The adult gynogenic diploids (line designated as TIFR) carry only the maternal genome and do not have cardiac mCherry fluorescence as the paternal genome was destroyed during haploid generation (Fig. 8J–L).

We analyzed the heat shocked gynogenic haploids at 24 hpf for morphologically normal embryos, which were then raised to assay for absence of haploid syndrome during development and inflation of swim bladder by 6–7 dpf. We chose swim bladder inflation as the first indicator of survival due to diploidization as this developmental landmark occurs very late in larval development and is an obligate requirement for larvae to transition into free swimming feeding fish, which haploids never transition to.

In four independent experiments, haploids heat shocked at 12, 18, and 24 mpf successfully transitioned into gynogenic diploids, which were morphologically indistinguishable from control diploid embryos with inflated swim bladders (Fig. 8F). However, the gynogenic diploids generated by transient heat at 18 mpf transitioned to the swim bladder stage significantly better, while those generated by 24 mpfHS showed a strong trend for this transition than those generated by transient heat shock at 12 mpf (Fig. 8M and Heier et al., 2015). The successful transition to swim bladder stage was 39% (n = 46/118) for 12 mpfHS, 59% (n = 24/41) for 18 mpfHS (chi‐square statistic 4.72 and *P*‐value 0.03 when compared with 12 mpfHS) and 40% (n = 30/75) for 24 mpfHS (chi‐square statistic 0.02 and *P*‐value 0.88 when compared with 12 mpfHS).

We next assayed if the gynogenic diploids that transitioned into larvae with swim bladders would demonstrate differential survival to adulthood based on the time of heat shock used for diploidization. From the four independent experiments, we find that gynogenic diploid larvae with swim bladders obtained from 12 and 18 mpfHS were equivalent in their potential to survive to adulthood (24% for 12 mpfHS, n = 11/46 and 29% for 18 mpfHS, n = 7/24, chi‐square statistic 0.23 and *P*‐value 0.63). However, in comparison with 12 mpfHS, gynogenic diploid larvae with swim bladders obtained from 24 mpfHS demonstrate a trend of poor survival to adulthood (10%, n = 3/30, chi‐square statistic 2.34 and *P*‐value 0.13).

Thus, the transient teratogenic heat resilient developmental windows at 18 mpf and 24 mpf could be useful in expediting phenotype driven recessive mutation screens up to ∼5–7 dpf. However, if the goal is to generate gynogenic diploid adults, a transient heat shock at 18 mpf may be preferred.

## Discussion

The ∼70% lethality even in embryos that undergo normal cytokinesis geometries and cellularization before epiboly after heat shocks indicate that transient heat is a potent teratogen during zebrafish embryonic development. Our experiments reveal that during key developmental times, zebrafish embryos are less susceptible to the teratogenic effects of transient heat shocks. We also show that such teratogenic heat resilient developmental windows are advantageous for generating gynogenic diploids. Our work reveals that two teratogenic heat resilient windows exist before the first zygotic mitosis in zebrafish: one is at the end of pronuclear congression and fusion phase and the second one during metaphase of mitosis‐I. Both can be used to optimally generate gynogenic diploids in comparison to 12 mpfHS, which is the original heat shock paradigm for genome doubling in zebrafish. The second teratogenic heat resilient window during metaphase of mitosis‐I is in agreement with heat shock 2 at 22 mpf that was recently characterized by Heier et al., 2015. The variation in our experiments to 24 mpf from the 22 mpf window reported by Heier et al., 2015 could be because the progression of mitosis and occurrence of metaphase‐I in the two studies is unlikely to be identical. A preliminary analysis similar to that done in Figure 3 should be undertaken to identify the phase of mitotic progression.

Our results suggest that the major window of resilience to teratogenic heat is at the end of pronuclear congression and fusion phase at 18 mpf with a second minor resilience window occurring during metaphase of mitosis‐I at 24 mpf. The fact that embryos at the end of pronuclear congression and fusion phase and at metaphase‐I specifically withstand teratogenic heat show that acquisition of teratogenic heat resilience is not a developmental transition rather it is a transient state of early embryonic development when the system is comparatively less susceptible to heat. We began our cell biology analysis in heat shocked zebrafish embryos with the view that a differential extent of perturbation and recovery response of the dynamic cell biology during earliest stages of development may underlie the differential survival observed in 18 and 24 mpfHS in comparison with 12 mpf. The immediate effect of heat is a perturbation of microtubule cytoskeleton and pericentrosomal organization. This is reminiscent of specific centrosomal damage seen in febrile patients, where the fever triggers centrosome degradation (Vertii et al., 2015).

Heat also affects centrosome duplication; however, the presence of two centrosomal foci in 12 mpfHS strongly suggest that the first round of centrosome duplication occurs before 12 mpf and cannot be perturbed in any of the heat shocks performed in this study. Centrosome duplication cycles are coupled to DNA duplication, which is evolutionarily conserved in eukaryotes (Fu et al., 2015). Therefore, the first round of DNA duplication in the zebrafish zygote most likely occurs in individual pronuclei, before 12 mpf. The 2C stall embryos complete mitosis‐I despite the heat shock and the failure in the second centrosome duplication cycle is evident as prophase‐II originally initiates with a single centrosome, while the second DNA duplication cycle has already occurred. In this category, mitosis‐II cannot proceed and because NEBD cycles are unperturbed, the third DNA duplication cycle occurs. During the third DNA duplication cycle, centrosomes also duplicate enabling resumption of mitosis. The 1C stall embryos enter mitosis‐I late (12 and 18 mpfHS) or re‐enter mitosis‐I after mitotic spindle disassembly upon heat shock (24 mpfHS). In this category, the second cycle of DNA duplication occurs before mitosis‐I, but centrosomes do not duplicate. Thus, in the 1C stall category as well the heat shock uncouples the DNA duplication from the second centrosome duplication cycle.

Twelve, 18, and 24 mpfHS all uncouple DNA duplication from the second centrosome duplication cycle, which recovers in the next mitosis. The heat does not extensively damage the embryos, as NEBD dynamics remain unaffected. The second centrosome duplication cycle necessitates the usage of a mother centriole template made entirely maternally for the first time in each cell of the zygote. It is intriguing that, upon heat shock, the ability to execute this milestone, which typically occurs in less than 5 min is compromised in the zygote. It is also intriguing that though the cell biological consequence of heat shocks are similar in 12, 18, and 24 mpfHS, survival is better and teratogenic effects of heat are significantly lower in 18 mpfHS.

The teratogenic heat resilient window of 18 mpfHS is particularly useful in generating adult gynogenic diploids in comparison to 12 mpf or 24 mpfHS. However, if the purpose of the diploidization strategy is to instantaneously homozygoze recessive loci in the gynogenic diploids, both 18 and 24 mpfHS could be used. In such experiments, the yield of gynogenic diploids obtained is influenced by the age of the female and the frequency with which a female is used for manual egg extrusions. In this study, we have used 5‐ to 12‐month‐old females and rested the female fish for 10–15 days between successive egg extrusion procedures.

In summary, we postulate that heat shocks during 12 to 26 mpf perturbs a state(s) in the developing zebrafish embryo, which transiently does not exist at 18 mpf, a brief period between end of pronuclear fusion and beginning of zygotic mitosis. This transient state could be physical connections between macromolecular structures, epigenetic state of the zygotic genome or metabolic states of the embryo and warrants further detailed investigations. For example, it has been shown that hypoxia lowers the temperature at which embryonic and organismal lethality occurs and hyperoxia enhances thermotolerance (Portner, 2002; Portner and Knust, 2007; Gendelman and Roth, 2012; Verberk et al., 2013; Smith et al., 2015). It may be interesting to connect the enhanced survival and reduced heat teratogenicity to mitochondrial divisions in the developing zebrafish embryo during the heat shocks, perhaps. Regardless of the mechanism, such transient teratogenic heat resilient windows point to the existence of innate states in metazoan embryos, which can confer protection from environmental insults during a phase of development when protective molecular responses cannot be evoked.

## Experimental Procedures

### Fish Husbandry

Standard laboratory strains of *Danio rerio* Tubingen and AB were raised in 14/10 hr of light/dark cycle. Embryos were raised in embryo medium in an incubator at 28 °C.

### IVF

All experiments were performed with temporally synchronized zebrafish embryos obtained by IVF. Adult males were euthanized and whole testes harvested into 800 μl of chilled Hank's buffer. Testes were macerated using a 1‐ml pipette and sperm solution was allowed to settle on ice. Adult females were primed for egg extrusion by natural pair matings overnight. As the females released the first wave of eggs, they were segregated from the males for further manual extrusion of eggs. Aproximately 150 eggs were extruded onto a clean, dry petri dish and fertilized with 100 μl of sperm solution. A drop of embryo medium was added to activate the eggs and after 1 min the plate was flooded with embryo medium. For all experiments, 5‐ to 12‐month‐old females were used and the female fish were rested for 10–15 days between successive manual egg extrusions.

### Heat Shocks

The conical bottom of a 50‐ml plastic tube was cut off and a hole made in the screw cap to hold a 0.8‐mm plastic mesh when the cap was in place on the tube. This was used as a submersible container for all heat shocks. Two 250‐ml beakers containing embryo medium were held in a water bath at 28 °C and 42 °C. Approximately 50 in vitro fertilized embryos were transferred into the heat shock tube and kept in the glass beaker at 28 °C until time of heat shock. For heat shocks, the heat shock tube was moved quickly into the 42 °C embryo medium for 2 min and back into the 28 °C embryo medium to quench the heat shock.

### Gynogenic Haploid Production

Testes were harvested from adult males and macerated in 800 μl Hank's solution on ice. The solution was allowed to settle for 10 min on ice and 200 μl of the supernatant was transferred to a watch glass and irradiated with UV light of 254 nm for 90 sec. Irradiated sperm solution was used to fertilize eggs from females to obtain gynogenic haploid embryos.

### Gynogenic Diploid Production

Gynogenic haploid embryos were subjected to transient heat shocks at 12 mpf, 18 mpf, and 24 mpf as described in the heat shock section. Embryos were scored at 24 hpf for normal morphology and again scored at 5–7 dpf for an inflated swim bladder and raised to adults. The gynogenic diploid line obtained in this study has been designated as TIFR. To score for lack of paternal trait inheritance in gynogenic diploids, UV irradiated sperm from *Tg(buc:TagRFP‐Has.TUBA1B, cmlc2:mCherry)* males were used for haploid production and then heat shocked at 12 mpf, 18 mpf, and 24 mpf. The transgenic line was obtained from National Bio Resource Project (NBRP) Japan and carries the *Tg(cmlc2:mcherry*) (Palencia‐Desai et al., 2011) insertion, which begins expressing at 1 dpf and continues in adults. Embryos were scored at 24 hpf for normal morphology, at 5–7 dpf for an inflated swim bladder and at ∼2.5 months for absence of cardiac mCherry in gynogenic diploid adults.

### Live Analysis of Cell Division Geometries

Heat shocked embryos and controls were periodically observed under the microscope for the first 90 mpf. At 35–45 mpf, when controls typically transition to the two‐cell stage, one‐cell heat shocked embryos were sorted into a separate petridish. From this dish, embryos that became two‐cell at 50–60 mpf when controls became four cells were sorted as 1C stalls into a new dish. At 35–45 mpf, heat exposed embryos that became two‐cells like controls were sorted into a second petridish. From this petridish, at 50‐60 mpf embryos that remained as two‐cells were sorted as 2C stalls, when controls became four cells. All heat shocked embryos, including from 1C and 2C stalls were monitored for abnormal cell division geometries and acellularized patches and kept separate. From the 1C and 2C stalls, embryos that underwent normal cell division geometries and normal blastoderm cellularization at 4 hpf were raised at 28 °C for survival and morphology analysis.

### Analysis of Developmental Progression and Survival

At 24 hpf, morphology of the sorted heat shocked embryos was compared with controls. Embryos that looked exactly like controls were categorised as “Normal” and embryos that looked different from controls, with a range of abnormalities, were classified as “Abnormal.” Embryos in each category were recorded. Statistical analysis was performed in Microsoft Excel and GraphPad Prism 5.0.

### Metaphase Chromosome Spread

Metaphase chromosome spreads were prepared as described in (Westerfield, 2000). Briefly, 24–30 hpf larvae were manually dechorionated and incubated in 100 µl of 4 mg/ml Colchicine at 28 °C in dark for 5 min. Larvae were transferred to 300 µl of 4 mg/ml Colchicine and incubated at 28 °C for 90 min in dark. Embryos were rinsed thoroughly with embryo medium without methylene blue and then transferred to 1 ml of 1.1% sodium citrate. The yolks of a few embryos were punctured in a span of 8 min at room temperature followed by incubation on ice for an additional 8 min. The sodium citrate was discarded and embryos were fixed in freshly prepared 3:1 mixture of methanol:acetic acid overnight at 4 °C. Embryos fixed overnight were transferred to a watchglass and the fixative was removed by blotting. Embryos were minced using forceps in a solution of 50% acetic acid and triturated using a 50 µl wiretrol capillary pipette to generate a single cell suspension. A total of 2–3 drops of this suspension were dropped onto clean slides prewarmed at 65 °C followed by incubation of slides at 65 °C for 60 min. The spreads were stained with DAPI in an antifade mounting medium (Vector Laboratories or Invitrogen) and allowed to dry overnight at room temperature. Chromosomes were imaged on Zeiss Axio‐Imager M2 and counted using Image J software.

### Immunofluorescence and Live Imaging

Embryos were fixed using 4% paraformaldehyde+2.5% glutaraldehyde fixative or 4% paraformaldehyde (for NPC). Immunolabeling was done as described previously (Pelegri et al., 1999). Primary antibodies used were mouse anti‐α‐tubulin (Sigma T5168, 1:2,500), rabbit anti‐γ‐tubulin (Sigma‐T3559, 1:2,000) and mouse monoclonal antinuclear pore complex proteins antibody Mab414 (Abcam‐ab24609, 1:1,000). Fluorescent secondary antibodies used were donkey anti mouse Alexa 555 and donkey anti rabbit alexa 488 (Invitrogen, 1:100). DNA was labelled with DAPI (Roche, 1:500 dilution of 0.5 µg/ml). Embryos were semi‐flat mounted and imaged on a Zeiss exciter, Zeiss LSM 510, or Olympus FV1200. Images were analyzed using ImageJ and assembled using AdobePhotoshop. For live imaging, embryos were dechorionated manually and mounted in 0.5% low melting agarose on depression slides. A drop of 1 × E3 was added to keep the embryo hydrated and images were acquired using differentiation interference contrast settings on Zeiss Axio Imager M2 microscope using Zen 2008 software.
